# Improved Dietary Guidelines for Vitamin D: Application of Individual Participant Data (IPD)-Level Meta-Regression Analyses

**DOI:** 10.3390/nu9050469

**Published:** 2017-05-08

**Authors:** Kevin D. Cashman, Christian Ritz, Mairead Kiely, ODIN Collaborators

**Affiliations:** 1 Cork Centre for Vitamin D and Nutrition Research, School of Food and Nutritional Sciences, University College Cork, Cork T12 Y337, Ireland; 2 Department of Medicine, University College Cork, Cork T12 DFK4, Ireland; 3 Department of Nutrition, Exercise and Sports, Faculty of Science, University of Copenhagen, Frederiksberg C DK-1958, Denmark; ritz@nexs.ku.dk; 4 Irish Centre for Fetal and Neonatal Translational Research (INFANT), University College Cork, Cork T12 DFK4, Ireland; m.kiely@ucc.ie

**Keywords:** vitamin D recommendations, DRV, RDA, EAR, Individual Participant Data-level meta-regression analyses

## Abstract

Dietary Reference Values (DRVs) for vitamin D have a key role in the prevention of vitamin D deficiency. However, despite adopting similar risk assessment protocols, estimates from authoritative agencies over the last 6 years have been diverse. This may have arisen from diverse approaches to data analysis. Modelling strategies for pooling of individual subject data from cognate vitamin D randomized controlled trials (RCTs) are likely to provide the most appropriate DRV estimates. Thus, the objective of the present work was to undertake the first-ever individual participant data (IPD)-level meta-regression, which is increasingly recognized as best practice, from seven winter-based RCTs (with 882 participants ranging in age from 4 to 90 years) of the vitamin D intake–serum 25-hydroxyvitamin D (25(OH)D) dose-response. Our IPD-derived estimates of vitamin D intakes required to maintain 97.5% of 25(OH)D concentrations >25, 30, and 50 nmol/L across the population are 10, 13, and 26 µg/day, respectively. In contrast, standard meta-regression analyses with aggregate data (as used by several agencies in recent years) from the same RCTs estimated that a vitamin D intake requirement of 14 µg/day would maintain 97.5% of 25(OH)D >50 nmol/L. These first IPD-derived estimates offer improved dietary recommendations for vitamin D because the underpinning modeling captures the between-person variability in response of serum 25(OH)D to vitamin D intake.

## 1. Introduction

Using the threshold for vitamin D deficiency of a serum 25-hydroxyvitamin D (25(OH)D) concentration <30 nmol/L [1] acknowledged as a primary risk factor for nutritional rickets in children [2] and osteomalacia in adults [3], a recent analysis from the ‘Food-based solutions for optimal vitamin D nutrition and health through the life cycle’ (ODIN) project, representing >55,000 individuals, reported that one in eight European residents has vitamin D deficiency [4]. Many investigators in the vitamin D field concur with the Endocrine Society’s 25(OH)D threshold of <50 nmol/L to designate vitamin D deficiency for protection of skeletal and non-skeletal health [5]. Thus, on the basis of the World Health Organization’s criteria [6] and the 25(OH)D threshold (30 or 50 nmol/L) selected, vitamin D deficiency in Europe can be classified as a mild (5%–19.9%) or severe (>40%) public health problem. Thus, strategies for vitamin D deficiency prevention are urgently required [7]. 

Dietary recommendations for vitamin D, hereafter referred to as dietary reference values (DRVs), have a key role in defining the parameters for such prevention strategies. In line with scientific developments, DRVs for vitamin D have been re-evaluated over the last six years [7,8]. Most agencies selected musculoskeletal outcomes as the health status indicators with a developed evidence base, particularly from randomized controlled trials (RCTs), on which to nominate serum 25(OH)D thresholds designating deficient/sufficient vitamin D status. Following analysis of vitamin D intake–serum 25(OH)D dose-response data, these thresholds provided the targets for estimating population and/or individual vitamin D intake recommendations [1,9,10,11,12]. All of the agencies based their analyses for vitamin D requirements assuming minimal ultraviolet B (UVB) exposure, as sunlight synthesis would interfere with the vitamin D intake–25(OH)D dose-response relationship. However, despite considerable effort by five different agencies over a 6-year period, the published DRVs are diverse. Individual recommendations vary from 10 to 20 µg/day (400–800 IU), depending on the 25(OH)D target, ranging from 25 to 50 nmol/L.

In the UK, the Scientific Advisory Committee on Nutrition (SACN) proposed a ‘population protective’ serum 25(OH)D concentration of 25 nmol/L, defined as the minimum threshold that should be met or exceeded by almost everyone, and recommended a corresponding vitamin D intake of 10 µg/day for all persons >1 year of age [9]. Four other agencies based their individual recommendations on achieving a serum 25(OH)D threshold ≥50 nmol/L [1,10,11,12], with some important distinctions. The Institute of Medicine (IOM) [1] and Nordic Council of Ministers (NORDEN) [11] recommended vitamin D intakes of 15 and 10 µg/day, respectively, for children and adults, with increased allowances for older adults. These two agencies also proposed an Average Requirement (AR) intake of 10 and 7.5 µg/day, respectively, to meet the needs of 50% of the population. The AR value is not an individual recommendation but used by nutritional epidemiologists to evaluate the adequacy of vitamin D intakes in the population [13]. The German Nutrition Society [12] and, more recently, the European Food Safety Authority (EFSA) [10] opted for Adequate Intake (AI) values for vitamin D of 20 and 15 µg/day, respectively, with the 50 nmol/L threshold in mind. The AI is reserved for use only in cases of much uncertainty in the data. 

While the serum 25(OH)D threshold selected will influence dietary requirement estimates for vitamin D, it is only one contributor to the disparity in recently published DRVs. An unintended but serious cause of this disparity is the method used to perform the vitamin D–25(OH)D dose-response analysis: use of a standard meta-regression approach based on aggregate data from several RCTs [1,10,11] versus use of individual data from distinct RCTs [9,12]. Apart from the impact of analytical differences in 25(OH)D values in different RCTs [14], there are pros and cons to both approaches. The meta-regression of aggregate data, which uses information averaged across all individuals within treatment groups in a RCT, can allow for between-study variability, but it cannot incorporate between-participant variability, which is crucial for estimating individual recommendations. A meta-regression based on analysis of individual participant data (IPD), in which the raw data for each RCT are used for synthesis, has many potential advantages, both statistically and clinically, over meta-regression of aggregate RCT data [15]. Modelling strategies for pooling of individual subject data from cognate dose-response RCTs are likely to provide appropriate estimates of DRVs to meet specified 25(OH)D thresholds, as use of individual data permits estimation of requirement values that cover 97.5% of the population group being considered. A recent Cochrane paper has highlighted that IPD analyses are not only now described as the gold standard, but that their use by guideline developers could lead to improved guidelines, ensuring that routine patient care is based on the most reliable evidence available [16]. 

Our hypothesis is that the method used to perform the dose-response vitamin D-25(OH)D analysis, on which DRVs are based, has a profound effect on the recommendation issued, regardless of the serum 25(OH)D target selected or other sources of heterogeneity. Thus, the objectives of this study were firstly to perform an IPD meta-regression using individual subject data (*n* = 882) from seven selected winter-based RCTs of the vitamin D intake–serum 25(OH)D dose-response, where raw data were available to the authors [17,18,19,20,21,22,23], in order to establish recommendations for vitamin D. Secondly, we wished to contrast these IPD-derived results against results from a standard meta-regression based on aggregate data derived from the same RCTs.

## 2. Materials and Methods, Including Scientific Approach

### 2.1. Context and Parameters of the Analyses

Our hypothesis is that the method used to perform the dose-response vitamin D-25(OH)D analysis, on which DRVs are based, has a profound effect on the recommendation issued, regardless of the serum 25(OH)D target selected. As our intention in this work is not to query or debate the 25(OH)D targets defined by agencies as part of the hazard identification step of the DRV risk assessment framework, we have applied our hypothesis to all four serum 25(OH)D thresholds (25, 30, 40, 50 nmol/L) that have featured in these reports [1,9,10,11]. IOM defined persons at increased risk of deficiency at a serum 25(OH)D <30 nmol/L, specifying 40 and 50 nmol/L as consistent with the requirements of 50% and 97.5% of individuals aged >1 year, respectively, for maintenance of bone health [1]. As 25 nmol/L is a widely-used cut-off in Europe, and was recently retained by SACN [9] to define vitamin D deficiency on the basis of metabolic bone disease, this was also included. While the Endocrine Society has suggested that serum 25(OH)D should exceed 75 nmol/L [5] to maximize the effect of vitamin D on calcium, bone, and muscle metabolism, <15% of study participants achieved >75 nmol/L during winter because of the maximum supplemental dose of 20 µg/day of vitamin D. Inclusion of this cut-off would have relied heavily on extrapolation, which we wished to avoid in the analysis. 

These analyses were conducted without consideration of the risk management implications of the outcomes, in terms of implementation of fortification or supplementation programs, as this step resides outside of the vitamin D DRV risk assessment framework process and the scope of this analysis. 

The DRI exercises undertaken by the IOM [1] and NORDEN [11] were based on collections of RCTs in which the age range of participants was 6–85 years. To explore whether the same DRV value would apply across the age-range, NORDEN included data from a repeated cross-sectional study of the elderly to augment the limited data from winter-time RCTs in older adults [11]. EFSA included 35 RCTs of children and adults and of the 83 treatment groups within these studies (i.e., RCT arms), nine arms were in children (age range: 2–17 years) [10]. EFSA included all RCT in the primary analysis and repeated the analysis excluding the data in children to quantify the impact on the estimates [10]. 

Finally, we wished to align with the approach adopted by the various agencies briefed with updating their DRV for vitamin D, namely one that prioritizes the identification of the intake values to maintain serum 25(OH)D concentrations above chosen cut-offs when UVB-induced dermal production of vitamin D is absent or markedly diminished. 

### 2.2. Measurement of Serum 25-hydroxyvitamin D

For three of the seven RCTs [17,19,22], concentrations of total 25(OH)D (i.e., 25(OH)D_2_ plus 25(OH)D_3_) in all serum samples were measured at the laboratory of the Cork Centre for Vitamin D and Nutrition Research using a Liquid chromatography–tandem mass spectrometry (LC-MS/MS) method that has been described in detail elsewhere [24,25] and is certified by the Centers for Disease Control and Prevention’s (CDC) Vitamin D Standardization Certification Program [26] and monitored on an on-going basis by participation in the Vitamin D External Quality Assessment Scheme (Charing Cross Hospital, London, UK). The intra-assay coefficient of variation (CV) of the method was <5% for all 25-hydroxyvitamin D metabolites, while the inter-assay CV was <6%. 

For another three RCTs [20,21,23], bio-banked sera, originally measured for serum 25(OH)D by enzyme-immunoassay (EIA), which has a positive bias [24], was reanalyzed for the present work by our LC-MS/MS method. Human serum and plasma 25(OH)D metabolites are stable when stored frozen for >10 years [27]; our samples were bio-banked at −80 °C and aliquots were not previously thawed. One RCT [18], in which serum 25(OH)D was originally measured by a high performance liquid chromatography (HPLC) method, did not have sufficient bio-banked sera for LC-MS/MS re-analysis of all samples, however, the original HPLC-derived 25(OH)D values were standardized to that of LC-MS/MS by targeted re-analysis of 69 samples spread across the serum 25(OH)D concentration distribution and application of the resulting calibration equation in the full data set (LC-MS/MS-derived 25(OH)D = 1.002 × (HPLC-measured 25(OH)D) + 6.145; *r*^2^ = 0.89).

### 2.3. Assessment of Total Vitamin D Intake

A quantitative, interviewer-administered food frequency questionnaire (FFQ) for vitamin D, which was developed and validated at the Cork Centre for Vitamin D and Nutrition Research, was used to estimate habitual vitamin D intakes from diet [28]. Individual estimates of vitamin D intake from the diet plus the doses administered in the RCTs delivered total vitamin D intake estimates. Each of the development and refinement steps central to the successful implementation of this FFQ were followed for each RCT implemented in the current analysis.

### 2.4. Meta-Regression Analysis of the Vitamin D–Serum 25-(OH)D Relation Using Data from the Seven Prioritized RCTs: Aggregate v. IPD-Based 

The conditional distribution of serum 25(OH)D at specific values of vitamin D intake was modelled assuming a linear relationship using three different meta-regression approaches: a two-step IPD meta-regression, a one-step IPD meta-regression, and a standard meta-regression based on aggregate data [15,29] (see detailed explanation in the next paragraph below). Following Stewart et al. [30], the two-step IPD meta-regression approach was prioritized as the most appropriate because of the modelling flexibility achieved by not making any simultaneous assumptions across RCTs. The two-step IPD meta-regression was carried out both as an unadjusted analysis (simple linear regression per study) and as two adjusted analyses (Analysis of Covariance (ANCOVA) per study) including the covariates age and baseline serum 25(OH)D.

The two-step IPD analysis proceeds as follows: In the first step, estimates of intake, predicted serum 25(OH)D level, and their lower 95% confidence or (participant-specific) prediction intervals as well as standard errors of all these estimates were obtained separately for each study. In the second step, estimates were combined and subjected to a standard random effects meta-regression analysis with the standard errors of the estimates as inverse weights [15]. Specifically, for each collection of estimates (i.e., estimated intakes or predicted serum 25(OH)D levels) from the separate analyses per study, a standard meta-regression model was fit. The one-step IPD meta-regression analysis was carried out as ANCOVA on pooled data from all seven RCTs, including baseline serum 25(OH)D and age as covariates. Finally, the standard random effects meta-regression analyses based on aggregate data were carried out both as unadjusted and adjusted analyses, in the latter case, including mean baseline serum 25(OH)D and mean age. In this approach, the standard error of the means was used as a proxy for between-subject variability. 

For all meta-regression models, estimation of required vitamin D intakes to maintain 50%, 90%, 95%, and 97.5% of the participants above serum 25(OH)D thresholds of 25, 30, 40, and 50 nmol/L (where feasible) was achieved by post hoc inverse regression on the lower limits of prediction intervals in the case of IPD analyses or confidence intervals in the case of standard meta-regression, respectively. Standard errors and 95% confidence intervals on these derived estimates were obtained as percentile intervals using non-parametric bootstrap with 1000 replications, as described previously [18]. All analyses were conducted using *R* version 3.3.2 (R Core Team, Vienna, Austria) and the R extension package “metafor” [31].

### 2.5. Sensitivity Analyses: Two-Step IPD

Body mass index (BMI): BMI has been inversely associated with 25(OH)D [32]. The mean BMI in the adult RCTs ranged from 26.1 to 28.9 in the present analyses. Neither IOM nor NORDEN included BMI in their models [1,11], and while EFSA tested an effect of BMI, it was not included as a covariate in their final model [10]. We included BMI as an additional covariate in a separate analysis in data from the four adult RCTs. 

Age: The IOM found no evidence of an age-effect on the response of serum 25(OH)D to increasing vitamin D intake and therefore included summary data from RCTs in both children and adults within their total data set [1]. NORDEN likewise combined data from RCTs in children and in adults [11], while EFSA tested the impact of restricting the data set to only adult RCT arms and excluding those from children. EFSA concluded that the overall estimates did not significantly change compared to the full data set including children and accordingly retained data on children and on adults in their analyses [10]. It is possible that age, as a surrogate for body size, may impact on the DRV estimates, and in fact EFSA suggested that children tended to achieve the same mean serum 25(OH)D concentrations as the adults at a lower total intake [10]. Thus, to further gauge the impact of age, an additional two-step IPD analysis adjusting for age and baseline serum 25(OH)D was carried out based on data from the four RCTs comprising adult participants (i.e., excluding studies on children/adolescents). 

Compliance: While subjects included in the analysis had all exceeded the minimum compliance threshold of 80%–85%, depending on the RCT, we tested whether increasing the compliance threshold limit to ≥95% potentially removed some of the variability in the data set in terms of response of serum 25(OH)D to a particular intake. We repeated the two-step IPD meta-regression analyses of the adult-only RCTs limited to subjects who met or exceeded a minimum of 95% compliance. These additional analyses, which removed 72 individuals from the data set, showed minimal alteration in estimates; there was only 0.1 and 1.4 µg/day lower difference in the estimates to maintain 97.5% of individuals with serum 25(OH)D >30 and >50 nmol/L, respectively, when these lower compliant individuals were removed (data not shown). Therefore, we thus decided to retain the full data set.

## 3. Results

A collection of seven RCTs, where raw data (*n* = 882 individuals) were available to the authors, was included in the present analysis. These RCTs were conducted in: 4–8 year-old children [17], 11 year-old girls [18], 14–18 year-old adolescents [19], adults aged 20–40 years [20], 50+ years [21,22], and 65+ years [23], and were all implemented using the same study design, analytical platform for serum 25(OH)D, and dietary assessment method. Most of these RCTs were among the 44 used collectively in the IOM, NORDEN, and EFSA exercises for deriving DRVs [1,10,11]. These seven RCTs all fulfill or exceed the previously defined RCT selection criteria established by the IOM as part of their process [1] (see Appendix A), and in fact five of the seven [18,20,21,22,23] have been used in the recent DRV meta-regression exercises by IOM, NORDEN, and EFSA [1,10,11]; the two most recent RCTs [17,19] were published since these. A brief description of each of the studies, including the sex and age of participants, the doses of vitamin D_3_, the published DRV estimate to prevent vitamin D deficiency (serum 25(OH)D < 25 nmol/L) in 97.5% of that population, and the trial registry numbers is provided in Table 1.

### 3.1. Vitamin D Requirement Estimates Based on the Two-Step IPD Meta-Regression Analyses

Based on the two-step IPD meta-regression model, the vitamin D intake estimates required to maintain serum 25(OH)D concentrations above the four serum 25(OH)D thresholds used by all of the regulatory agencies are shown in Table 2. Recommended Intakes, defined as the intake estimated to meet the requirement of 97.5% of the population for a specific 25(OH)D threshold, were as follows:

- Using the UK SACN 25(OH)D cut-off of ≥25 nmol/L (9), we estimated the vitamin D requirement to be 9.9 µg/day;
- The IOM, NORDEN, and EFSA used 30 nmol/L to indicate an increased risk of vitamin D deficiency [1,10,11], but they did not recommend an intake for this threshold. We estimated that the vitamin D requirement required to maintain 97.5% of individuals ≥30 nmol/L was 13.1 µg/day;
- The vitamin D intake requirement estimate allowing 97.5% of individuals to maintain serum 25(OH)D ≥50 nmol/L (the serum 25(OH)D threshold selected by IOM [1], NORDEN [11], and EFSA [10]) was 26.1 µg/day (Table 2).

Average Requirements (AR), defined as the intake estimated to meet the requirement of 50% of the population for a specific threshold, were as follows: 

Using the IOM 25(OH)D cut-off for the AR of 40 nmol/L [1], we estimated that the vitamin D intake required to maintain serum 25(OH)D concentrations ≥40 nmol/L in 50% of the subjects was 4.5 µg/day (Table 2). 

The AR meeting the NORDEN [11] serum 25(OH)D threshold of ≥50 nmol/L was 10.9 µg/day (Table 2). 

Table 2 shows that moving from the 90th percentile through the 95th to the 97.5th percentile of requirement increased the intake estimate dramatically; this is expected to meet the needs of ‘nearly all’ healthy individuals in the population (i.e., 97.5%). The equivalent vitamin D intake requirement estimates based on a regression model unadjusted for age and baseline serum 25(OH)D are also shown in Table 2, and for the most part were of the order of ~1–3 µg/day higher. 

**Figure 1:**
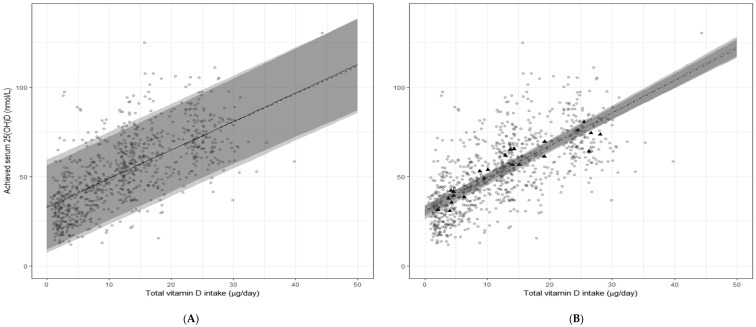
The relation between serum 25-hydroxyvitamin D (25(OH)D) concentrations (in late winter) and total vitamin D intake (i.e., from diet plus supplemental) in healthy persons aged 4–86 years living at northerly latitudes (between 51° N and 60° N) based on individual participant data (IPD) (*n* = 882 individuals) (**A**) and aggregate RCT group mean data (*n* = 23 arms) (**B**) from the same RCTs. The solid and dashed lines through the IPD data points (black circles) (**A**) correspond to the fitted regression lines based on the two-step (adjusted for age and baseline 25(OH)D) and one-step IPD analyses, respectively, and the corresponding 95% prediction bands are shown in grey (the lightest being the band for the one-step IPD analysis) (**A**). The fitted regression lines for the unadjusted and adjusted (age and baseline 25(OH)D) standard meta-regression based on aggregate data from same RCTs (black triangles) (**B**) are shown as dotted and dot-dashed lines with corresponding 95% confidence bands shown in grey (the lightest being the band for the unadjusted analysis).

Using a one-stage IPD approach, the Recommended Intake estimates (95% CI) were very close to those from the two-step IPD analyses: 10.8 (9.7, 11.8) and 27.0 (25.6, 28.5) µg/day using serum 25(OH)D thresholds of ≥25 and ≥50 nmol/L, respectively. Likewise, the AR estimates (95% CI) were similar to those from the two-step IPD analyses: 3.6 (2.7, 4.4) and 10.1 (9.5, 10.7) µg/day using serum 25(OH)D thresholds of ≥40 and ≥50 nmol/L, respectively. The similarity in estimates between the one-step and two-step approaches can be explained by the closeness of fitted regression lines for both (see Figure 1A), even though the two-step approach yielded a slightly narrower prediction band.

### 3.2. Outcomes of the Sensitivity Analyses for the Two-Step IPD

BMI: Inclusion of BMI as an additional covariate in the adult RCT dataset produced estimates to maintain 97.5% of individuals with serum 25(OH)D >30 and >50 nmol/L of 15.0 and 28.4 µg/day, respectively, compared to 15.0 and 28.7 µg/day, respectively, in the dataset unadjusted for BMI.

Age: The repeated two-step IPD meta-regression analysis in the four RCTs on adults only provided estimates to maintain 97.5% of individuals with serum 25(OH)D >30 and >50 nmol/L of 15.0 and 28.7 µg/day, respectively, compared to 13.1 and 26.1 µg/day, respectively, in the full data set.

### 3.3. Comparison with Vitamin D Requirement Estimates from Standard Meta-Regression Analyses Based on Aggregate Data

In order to fulfill our secondary objective, which was to perform standard meta-regression analyses based on an aggregate from the same RCTs used in the two-step IPD analysis, we extracted the data on the basis of each RCT arm. This yielded 23 separate RCT summary data points. The relation between achieved serum 25(OH)D concentrations and the total vitamin D intake (diet and supplemental) in the 23 arms of the seven RCTs using the standard meta-regression approach is shown in Figure 1B. In the standard meta-regression model of aggregate data from the seven RCTs (23 arms), adjusted for age and baseline serum 25(OH)D, the intake estimate (95% CI) required to maintain 97.5% of individuals ≥50 nmol/L serum 25(OH)D was 14.2 (10.1, 18.9) µg/day. The unadjusted model yielded a slightly higher estimate of 15.8 (11.0, 16.9) µg/day. The discrepancy in estimates arising from the IPD-based and standard meta-regression model of aggregate data can be summarized as a more biased regression line with a considerably narrower accompanying prediction band in the latter, whether based on unadjusted or adjusted analyses (Figure 1B).

While the best fit model in the present analyses was found to be a linear fit, which is in keeping when a plateauing effect at vitamin D intakes above 35 µg/day (1), some of the agencies have used a curvilinear (natural logarithmic (Ln)) model [1,10,11]. For the purposes of comparison, we also applied a curvilinear (Ln) model to the same seven RCT data set as used for the linear model. Using this model, the intake requirement estimates to maintain 97.5% of individuals ≥50 nmol/L serum 25(OH)D were 12.5 μg/day and 13.2 μg/day for adjusted and unadjusted models, respectively.

## 4. Discussion

DRVs for vitamin D established over the last 6 years are highly variable. This is a major concern in light of their crucial role in providing a framework for the prevention of vitamin D deficiency in the population and evaluating progress towards this goal, as well as providing guidance for clinical practice and for individuals. This study set out firstly to establish a proof of concept that an IPD meta-regression using individual subject data from selected winter-time RCTs of the vitamin D intake–serum 25(OH)D dose-response is a superior approach in the determination of recommendations for vitamin D, both at a population average and individual level, as it avoids some of the limitations intrinsic to standard meta-regression, based on aggregate data, as used in recent DRV estimates [1,10,11]. Secondly, we aimed to provide experimentally-derived DRV estimates that would achieve the range of serum 25(OH)D target values selected by the different international agencies. We conducted our analysis in a pooled sample of 882 participants, ranging in age from 4 years upwards, from seven dose-response vitamin D intervention studies, all implemented using the same design, during wintertime at locations >50° N and with data for the axes for the dose-response analysis (i.e., 25(OH)D concentrations and vitamin D intake produced using certified and validated methods, respectively). Using this data set, the present analysis clearly illustrates that the vitamin D DRV estimates arising from an IPD approach, increasingly recognized as best practice [15,16], were considerably higher than those derived from the standard meta-regression approach based on aggregate data. This was strikingly evident in the large gap in estimates for recommended intakes derived by the two-step IPD analyses (~26 µg/day) and by standard meta-regression (~14 µg/day) to maintain serum 25(OH)D ≥50 nmol/L in 97.5% of individuals, favored by most agencies [1,10,11,12].

The disparity arises, to a large extent, from the inability of the standard meta-regression approach (even in adjusted models) to recover between-individual variability from summary statistics (i.e., mean and standard error per arm), which is a critical short-coming in the context of deriving DRVs. The large inter-individual variation that exists in the response of serum 25(OH)D to any particular intake of vitamin D underlies the wide prediction intervals used to estimate the DRV intake estimates at the 97.5th percentile. At best, between-study variation may be recovered using such an approach [15], and we have shown in the present analysis that between-study variation does not account for between-individual variation. This is not unexpected, as they arise from two completely different sources of variation. It has been recently suggested that failure to assimilate information from IPD-based approaches may lead to limited recommendations that are inappropriate for population health. On the other hand, the opportunity afforded by their uptake in preference to standard aggregate approaches may better inform guidelines [16]. This study, by clearly demonstrating the significant advantage of using an IPD approach, adds further evidence to support its application to the process of deriving DRVs. 

There are wide differences between our DRV estimates and those from some of the agencies, as summarized in Table 3. The intakes recommended by IOM, NORDEN, and EFSA, intended to maintain serum 25(OH)D ≥50 nmol/L in 97.5% of individuals during wintertime, used for setting personal targets, or assessing individual intakes, at 10 µg/day (NORDEN) and 15 µg/day (IOM and EFSA) are much lower than our experimentally derived value of 26 µg/day. Due to the inability of the standard meta-regression approaches used by these three agencies to capture between-individual variability [1,10,11], these DRVs for vitamin D will not provide the level of population protection anticipated at the time of their establishment. The UK SACN established a Recommended Intake of 10 µg/day to maintain serum 25(OH)D ≥25 nmol/L in winter for 97.5% of the population [9], based on three separate analyses of individual subject level data from distinct age-group specific RCTs [18,20,23]. Our analysis, based on the two-step IPD, derived an experimentally derived intake of 9.9 µg/day to maintain serum 25(OH)D concentration ≥25 nmol/L in winter for 97.5% of the population, which in Table 3 is rounded up to 10 µg/day. 

To enable evaluations of the adequacy of vitamin D intakes on a population basis, two agencies proposed estimated ARs [1,11]. The IOM’s AR of 10 µg/day is based on maintaining serum 25(OH)D ≥40 nmol/L in 50% of individuals (aged 1 year old and upwards) during winter. Notwithstanding their declared uncertainty in their simulated dose-response relationship, the IOM used it, on the basis that this intake would considerably over-shoot the targeted serum 25(OH)D concentration [1]. Our IPD analyses, which is free of such uncertainty in the dose-response relationship due to its underpinning individual level analytical data, shows that an intake of 4.5 µg/day during winter will maintain serum 25(OH)D ≥40 nmol/L in 50% of individuals, aged 4–86 years. The AR estimates from the two types of models (IPD analyses v. standard meta-regression with aggregate data) are much closer than these figures might suggest at face value, and this is due to the fact that both are based on an average and do not rely on the ability to use between-individual variation, in contrast to Recommended Intake estimates. To illustrate this point, the IOM standard meta-regression model showed that an intake of 10 µg/day would give a projected mean serum 25(OH)D of ~52 nmol/L [1], while the present work based on IPD analysis shows that an intake of ~10 µg/day will maintain serum 25(OH)D ≥50 nmol/L in 50% of individuals (aged 4+ years) during winter. 

The impact of using our experimentally derived AR estimate of 4.5 µg/day versus the IOM value of 10 µg/day to compare the prevalence of inadequate intakes of vitamin D in the general population is striking. For example, 55% and 89% of participants in the National Adult Nutrition Survey in Ireland would have inadequate intakes of vitamin D using these two benchmarks, respectively [33]. Furthermore, the median intake of vitamin D in adults (aged 18–84 year) participating in the Irish adult nutrition survey was 3.7 µg/day [33], and the measured prevalence of serum 25(OH)D <40 nmol/L in winter was 47% [24]. The apparently contradictory evidence of a much higher prevalence of inadequate intakes compared with vitamin D status is therefore a function of the AR itself. This does not diminish the need to address vitamin D deficiency on a population-wide basis, but it does highlight the need to work towards well-founded DRVs for nutritional surveillance.

It has been stressed that IPD analyses are not without their challenges, including being resource intensive. The issue of limited availability of data for some studies could introduce bias [15]. In this study, biochemical re-analysis of samples was required to minimize a confounding effect of method-related differences in the outcome measure. In a wider context, this would have a knock-on impact on the availability of samples in addition to data from RCTs identified in the systematic review approach. Another option that has been suggested is to collaborate with other research groups and agree to pool resources to answer specific questions [16]. This is the approach adopted in this work which allowed us to secure serum samples for re-analysis of serum 25(OH)D and remove some of the method-related confounding that is likely intrinsic in DRV estimates to date.

A strength of the present analyses is the high-quality of the RCTs used, which were designed to address this specific question. The studies used in the present work all fulfill or exceed the previously defined RCT selection criteria established by the IOM [1], and five of the seven have been used in the recent DRV meta-regression exercises by IOM, NORDEN, and EFSA [1,10,11]. The two most recent RCTs in adolescents and children were published after the most recent reports. That the seven RCTs met with these predefined selection criteria while also minimizing serum 25(OH)D method-related differences ensured the data were of the highest quality for inclusion in an IPD analysis. It could be fairly argued that by striving to achieve a high degree of internal validity in our analysis by our selection of RCT that were all implemented using the same study design, analytical platform for serum 25(OH)D, dietary assessment method, and statistical approach, it may diminish the external validity of our findings and limit their generalizability to other contexts (i.e., other populations beyond those used in the included RCTs). However, the winter-based vitamin D RCT collection used in the present analysis is not substantially different in terms of a number of RCTs of children and adults than that used by IOM (*n* = 11; 1) or NORDEN (*n* = 6; 11), and all of which used winter-based RCTs performed above a latitude threshold of 49.5° N. EFSA, by choosing less strict inclusion criteria for their winter-based RCTs, and especially a much wider latitude range >40°N, utilized data from 35 RCTs [10]. We argue that the EFSA’s assumption of minimal UVB-induced synthesis would not be met in several of these RCTs based on the latest UVB availability data for Europe [34]. It is of note that the IOM, after performing their meta-regression in RCTs in the latitude band of 40–49.5° N, as well as in those conducted at >49.5° N, decided not to use those from the lower latitude band for this reason [1]. Despite this, it is of note that the unadjusted Ln-models performed by these three agencies [1,10,11] as well as ourselves, and especially in the face of the differences in RCT datasets, all yielded relatively similar mean achieved serum 25(OH)D to a total vitamin D intake of 10 µg/day (as an estimate all four exercises had included and thus allows for comparison), namely, in the range 52–55 nmol/L. This provides added confidence that the generalizability of our estimates is robust. In addition, the fact that the various sensitivity analyses performed in the present work (e.g., removal of RCT data from children, inclusion of BMI, increasing the compliance threshold to ≥95%) had no major impact on the findings further speaks towards the robustness of the estimates. This was also the case in the analyses by EFSA, in which age or BMI did not seem to impact on their modelling and thus were not included in their final model [10]. 

The present analyses were based on linear models of vitamin D intake–serum 25(OH)D relationship, not only as the best fit model, but also in line with the suggestion that only at intakes >35 µg/day does the response of serum 25(OH)D to vitamin D begin to plateau [35]. In terms of vitamin D intakes in the present analyses, only 0.3% of IPD data and none of the meta-regression summary intakes were >35 µg/day. This is also of relevance from a population perspective, where the 95th percentile of intakes of vitamin D in various European [36] as well as the US population [37] sit very well with the total vitamin D intake range in the present RCT dataset (1–44 µg/day). Despite these arguments, we recognize the fact that three of the agencies have applied curvilinear meta-regression models to the intake–status RCT data [1,10,11]. Importantly, our analyses show that the estimates to maintain 50 nmol/L from an adjusted Ln-based standard meta-regression model of aggregate data yielded relatively similar estimates as the adjusted linear-based equivalent (13.2 vs. 14.2 µg/day, respectively). 

Importantly, we would also stress that even inclusion of aggregate data from as many as 35 RCTs as per EFSA [10], while enhancing the representativeness and quantifying more precisely between-study variability, will not recover the between-person variability from within the datasets needed for providing an unbiased RDA-like estimate. Figure 1 in the present analysis demonstrates very clearly what is referred to as an ecological bias in the fitted standard meta-regression lines, reflecting that these models only picked up the relationship between vitamin D intake and serum 25(OH)D at the level of the studies considered, but not at the level of participants (these two relationships need not be the same) [38]. The IPD analyses do not have such a bias because they rely on participant-level data. 

Limitations in the present analysis stem from the type of individual participant data from appropriate RCTs available to us. While our age-range included individuals from 4 to 86 years, <5% of the sample were aged >75 years of age. This lower availability of data for the elderly subset of the population was also noted by NORDEN [11]. We also note the lack of data for children <5 years, which is an acknowledged research gap [39]. Access to current and accurate food composition data is a requirement for the estimation of vitamin D intakes, a key input into DRV modelling exercises. More comprehensive coverage of the vitamin D content, including 25(OH)D, of staple foods is required within food composition databases [40]. It should be noted, however, that the harmonized dietary assessment method used in all seven RCTs in the present analysis is based on food composition data from various sources to minimize such gaps. All data from animal-derived sources included estimates of potency-adjusted 25(OH)D as well as vitamin D [41].

## 5. Conclusions 

In conclusion, this first-ever IPD level meta-regression of the vitamin D intake–serum 25(OH)D dose-response suggests that the vitamin D intakes required to prevent vitamin D deficiency (<25 nmol/L) and inadequacy (<50 nmol/L) in 97.5% of the population (i.e., the RDA estimate) is 10 and 26 µg/day, respectively. These first IPD-derived estimates are considerably different from those of the agencies [1,10,11] that used a standard meta-regression, based on aggregate data, to analyze the vitamin D intake–serum 25(OH)D dose-response relationship, due to the inability of such standard meta-regression analyses to capture between person-variability.

## Figures and Tables

**Table 1:** Selected design parameters of the seven winter-based, North European (>50° N) vitamin D randomized controlled trials and baseline characteristics of subjects who completed the intervention studies and were included in data analyses.

Study	Cashman et al. 2008	Cashman et al. 2009	Cashman et al. 2011	Cashman et al. 2012	Cashman et al. 2014	Smith et al. 2016	Mortensen et al. 2016
(Reference Number)	[19]	[22]	[17]	[20]	[21]	[18]	[16]
Trial registry ID	ISRCTN20236112	NCT01990872	NCT00267540	NCT01398202	NCT01990872	ISRCTN40736890	NCT02145195
Design parameters							
Location (° N)	Ireland (51° N and 55° N)	Ireland (51° N and 55° N)	Denmark (55° N)	Ireland (51° N)	Ireland (51° N)	UK (51° N)	Denmark (55° N)
Year of study	2006–2007	2007–2008	2001–2002	2011	2012–2013	2014–2015	2014–2015
Duration (and Months)	22 weeks (October–April)	22 weeks (September–April)	22 weeks (October–April)	10 weeks (January–April)	15 weeks (November–March)	20 weeks (October–March)	20 weeks (September–March)
Supplemental vitamin D_3_ dose(s) (µg/day)	0, 5, 10, 15	0, 5, 10, 15	0, 5, 10	0, 20	0, 20	0, 10, 20	0, 10, 20
Subject characteristics:							
*n*	196	176	140	28	122	102	119
Sex (Male:Female)	98:98	70:106	140 F	13:16	34:88	86:130	55:64
Age (year)	29.9 ± 6.2 ^1^	70.7 ± 5.4	11.3 ± 0.3	57.2 ± 6.3	59.9 ± 6.5	15.9 ± 1.4	6.7 ± 1.5
Weight (kg)	77.0 ± 15.8	76.9 ± 4.3	42.1 ± 8.5	79.0 ± 15.3	72.5 ± 13.3	60.7 ± 12.7	23.4 ± 5.0
Height (m)	1.71 ± 0.009	1.62 ± 0.09	1.50 ± 0.07	1.67 ± 0.08	1.64 ± 0.11	1.70 ± 0.10	1.22 ± 0.10
BMI (kg/m^2^)	26.1 ± 4.3	28.9 ± 4.8	18.6 ± 3.0	28.3 ± 4.8	26.7 ± 4.2	21.3 ± 3.4	15.5 ± 1.4
Dietary calcium (mg/day)	976 (682, 1301) ^2^	874 (678, 1174)	1122 ± 582	971 ± 445	814 ± 413	853 (591, 1249)	683 (485, 914)
Dietary vitamin D (µg/day)	3.6 (2.1, 5.4)	4.4 (2.7, 5.9)	3.7 ± 2.0	5.4 (3.5, 8.2)	4.4 (2.9, 6.8)	3.3 (2.2, 5.5)	1.8 (1.2, 2.5)
Serum 25(OH)D (nmol/L)	66.1 ± 22.5 *	51.9 ± 18.6 *	62.8 ± 13.5 *	41.5 ± 15.7 *	55.1 ± 20.4	48.2 (41.2, 22.70)	56.7 ± 12.3
Published RDA at 25 nmol/L (µg/day) ^3^	8.7	8.6	8.3	NR	NR	10.1	6.4

BMI, body mass index; 25(OH)D, 25-hydroxyvitamin D; RDA, recommended dietary allowance, NR, not reported. * Based on LC-MS/MS reanalyzed or standardization, not by original method of analysis; ^1^ Mean ± SD (all such values); ^2^ Median (IQR) of non-normally distributed variable (all such values); ^3^ As reported in the original publications and with variation in the original analytical platforms for serum 25(OH)D across this collection of randomized controlled trials (RCTs).

**Table 2:** Individual Participant Data (IPD) level meta-regression-derived dietary requirements for vitamin D at selected percentiles to maintain serum 25(OH)D above selected concentrations during winter–adjusted and unadjusted models.

Serum 25(OH)D	50th Percentile ^3^	90th Percentile	95th Percentile	97.5th Percentile ^4^
**Adjusted model ^1^**	µg/day			
≥25 nmol/L	-	4.7 (3.6, 5.9)	7.5 (5.9, 9.1)	9.9 (7.9, 12.0)
≥30 nmol/L	-	7.9 (6.5, 9.2)	10.6 (8.8, 12.5)	13.1 (10.8, 15.4)
≥40 nmol/L	4.5 (3.1, 6.0)	14.2 (12.3, 16.2)	17.1 (14.6, 19.6)	19.6 (16.5, 22.6)
≥50 nmol/L	10.9 (9.2, 12.5)	20.7 (17.9, 23.5)	23.5 (20.2, 26.9)	26.1 (22.1, 30.1)
**Unadjusted model ^2^**	µg/day			
≥25 nmol/L	-	6.8 (5.0, 8.6)	9.9 (7.5, 12.3)	12.7 (9.7, 15.7)
≥30 nmol/L	-	9.7 (7.6, 11.8)	12.9 (10.1, 15.6)	15.7 (12.3, 19.0)
≥40 nmol/L	4.7 (3.3, 6.1)	15.7 (12.9, 18.5)	18.9 (15.4, 22.3)	21.7 (17.6, 25.8)
≥50 nmol/L	10.6 (8.7, 12.4)	21.6 (18.1, 25.2)	24.8 (20.6, 29.1)	27.7 (22.7, 32.6)

^1, 2^ Results based on a two-step IPD approach which related serum 25(OH)D concentration as a function of vitamin D intake for *n* = 882, which was adjusted for age (mean) and baseline serum 25(OH)D (mean) or unadjusted. 95% Confidence Intervals (CIs) for the lower prediction limits were obtained using bias-corrected bootstrap based on 1000 replications. EAR, Estimated average requirement; RDA, Recommended dietary allowance; 25(OH)D, 25-hydroxyvitamin D; ^3^ The vitamin D intake that will maintain serum 25(OH)D concentrations in 50% of individuals above the indicated cut-off concentration during winter, representing an EAR; ^4^ The vitamin D intake that will maintain serum 25(OH)D concentrations in 97.5% of individuals above the indicated cut-off concentration during winter, representing an RDA.

**Table 3:** Comparison of recent international DRVs with new empirical data from the present two-step IPD analyses using one of the best collections of RCT data available (total *n* = 882 individuals).

Name of Agency: Specified DRV	Criterion Appliedby Establishing Agency	Agency Recommendation (µg/day)	Our IPD-Derived Value (µg/day)
IOM: RDA	Intake to meet needs of 97.5% of individuals at target serum 25(OH)D of 50 nmol/L	15	26
NORDEN: RI	Intake to meet needs of 97.5% of individuals at target serum 25(OH)D of 50 nmol/L	10	26
EFSA: AI	Intake to meet needs of 97.5% of individuals at target serum 25(OH)D of 50 nmol/L	15	26
SACN: RNI	Intake to meet needs of 97.5% of individuals at target serum 25(OH)D of 25 nmol/L	10	10
IOM: EAR	Intake to meet needs of 50% of individuals at target serum 25(OH)D of 40 nmol/L	10	4.5
NORDEN: AR	Intake to meet needs of 50% of individuals at target serum 25(OH)D of 50 nmol/L	7.5	11

IOM, Institute of Medicine; EAR, Estimated Average Requirement; NORDEN, Nordic Nutrition Recommendations; AR, Average Requirement; RDA, Recommended Daily Allowance; RI, Recommended Intake; EFSA, European Food Safety Authority; AI Adequate Intake; SACN, UK Scientific Advisory Committee on Nutrition; RNI, Reference Nutrient Intake.

## Appendix

### Appendix A. Supplemental Materials and Methods

#### Predefined Criteria for Selection of Randomized Controlled Trials for Inclusion in DRV Meta-Regression Analyses

The following briefly outlines the predefined criteria used by the Institute of Medicine (IOM) in their 2011 Dietary Reference Intake exercise [1] in terms of selection of randomized controlled trials (RCTs) considered most appropriate to address the specific question of setting dietary requirements for vitamin D to meet pre-specified 25-hydroxyvitamin D (25(OH)D) thresholds. While Nordic Council of Ministers (NORDEN) [11] and, more recently, the European Food Safety Authority (EFSA) [10] used IOM’s criteria for the most part, there were some important points of difference in the inclusion criteria among the three agencies, which we will highlight below. All three agencies adopted an approach that prioritizes the identification of the intake values to maintain serum 25(OH)D concentrations above chosen cutoffs when UVB-induced dermal production of vitamin D is absent or markedly diminished.

The included studies in the DRV exercises were RCTs of vitamin D supplementation in apparently healthy humans or in patients (in which there was no underlying reason for altered vitamin D metabolism or response to vitamin D supplementation; also note as infants and pregnant or lactating women are life-stage groups that have special considerations in relation to vitamin D they were excluded from the meta-regression analyses) that fulfilled the following characteristics:

(1) Vitamin D_3_ administered orally on a daily basis, but limited to:
  - ≤1200 IU/day (30 µg/day; 1 µg = 40 IU) (NORDEN),
  - ≤2000 IU/day (50 µg/day) (IOM),
  - or ≤4000 IU/day (100 µg/day) (EFSA)

There are several RCTs which have used supplemental doses of vitamin D higher than even these thresholds (see review [42]), but such high intakes far exceed even the 95th percentile of intakes of vitamin D in most populations [36,37].

Inclusion of vitamin D_3_, not vitamin D_2_, was on the basis that the IOM Dietary Reference Intake (DRI) committee and EFSA used studies with vitamin D_3_ in their regression analyses, upon which DRI/DRV values were set [1,10], and, while there is still some debate, there is evidence that the relative potency of vitamin D_2_ in terms of raising serum total 25(OH)D is lower than that achieved with vitamin D_3_ [43]. The IOM only selected studies which provided vitamin D alone and not with co-administration of calcium, while NORDEN and EFSA included studies which co-administered calcium. As there is debate on whether calcium intake, particularly supplemental calcium, influences the response of serum 25(OH)D to vitamin D supplementation, we prioritized IOM’s approach over that of NORDEN and EFSA;

(2) No vitamin D metabolites [25(OH)D and 1,25(OH)2D] and analogues (e.g., alfacalcidol) co-administered;
(3) Winter-based studies, performed at relatively high latitudes to ensure minimal impact of UVB on the vitamin D intake–25(OH)D dose-response relationship and thus the calculated vitamin D intake requirements to achieve serum 25(OH)D thresholds. The selected latitude ranges/thresholds were:
  - >40° N (EFSA),
  - ≥49.5° N (or ≥49.5° S) (IOM),
  - or ≥50° N (NORDEN)

IOM included RCTs above 49.5° N only, as they showed that the requirement for vitamin D was smaller when data from RCTs performed in the latitude band 40–49° N were compared with those from RCTs at ≥49.5° N [1]. Recent European data on UVB availability shows that at 40–50° N, dermal production of vitamin D is possible in some winter months between October and April [34], which was specified by EFSA as the Winter period [10]. On the other hand, while IOM used RCTs performed at ≥49.5° N, some had end-dates in late Spring up to June, when dermal production of vitamin D is possible, thus not fulfilling the criteria of minimal UVB availability. Accordingly, we used a latitude threshold of ≥49.5° N and defined extended winter as November–March [1].

(4) Minimum of 6 weeks duration (on the basis that following initiation of vitamin D supplementation, serum 25(OH)D concentrations reach equilibrium after 6–8 weeks in adult and elderly subjects [44]). IOM did not apply this criteria as RCTs as short as 4 weeks were included;
(5) Reported serum or plasma 25(OH)D following supplementation in at least one intervention group and one control group;
(6) Assessment of vitamin D intakes on which to base the dose-response calculations. The IOM, NORDEN, and EFSA prioritized use of ‘total vitamin D intake‘ (i.e., vitamin D intake from diet as well as that from supplemental vitamin D dose used in the RCT) [1,10,11], which was the best overall estimate of vitamin D intake.

EFSA performed a risk of bias assessment on all RCTs they considered and assigned an overall risk of bias ranging from low to high [10].

### Appendix B. ODIN Collaborators:

Camilla T. Damsgaard and Christian Mølgaard, Department of Nutrition, Exercise and Sports, Paediatric and International Nutrition, Faculty of Science, University of Copenhagen, Denmark.

Christel Lamberg-Allardt, Department of Food and Environmental Sciences, University of Helsinki, Helsinki, Finland.

Kath Hart and Susan A. Lanham-New, Department of Nutritional Sciences, School of Biosciences and Medicine, Faculty of Health & Medical Sciences, University of Surrey, Guildford, Surrey, United Kingdom.
